# Development and Application of a High Throughput Protein Unfolding Kinetic Assay

**DOI:** 10.1371/journal.pone.0146232

**Published:** 2016-01-08

**Authors:** Qiang Wang, Nicklas Waterhouse, Olusegun Feyijinmi, Matthew J. Dominguez, Lisa M. Martinez, Zoey Sharp, Rachel Service, Jameson R. Bothe, Elliott J. Stollar

**Affiliations:** 1 Department of Physical Sciences, Eastern New Mexico University, Portales, New Mexico, United States of America; 2 NMRFAM, University of Wisconsin-Madison, Madison, Wisconsin, United States of America; University of Bath, UNITED KINGDOM

## Abstract

The kinetics of folding and unfolding underlie protein stability and quantification of these rates provides important insights into the folding process. Here, we present a simple high throughput protein unfolding kinetic assay using a plate reader that is applicable to the studies of the majority of 2-state folding proteins. We validate the assay by measuring kinetic unfolding data for the SH3 (Src Homology 3) domain from Actin Binding Protein 1 (AbpSH3) and its stabilized mutants. The results of our approach are in excellent agreement with published values. We further combine our kinetic assay with a plate reader equilibrium assay, to obtain indirect estimates of folding rates and use these approaches to characterize an AbpSH3-peptide hybrid. Our high throughput protein unfolding kinetic assays allow accurate screening of libraries of mutants by providing both kinetic and equilibrium measurements and provide a means for in-depth ϕ-value analyses.

## Introduction

The study of protein folding kinetics and stability is central to understanding protein structure, dynamics and energetics. Whereas global stability of a protein measures the proportion of a protein that is in its native state at equilibrium, folding rates provide information on protein’s kinetic stability. Kinetic stability is important to consider under physiological conditions of the cell where a protein in the unfolded state may irreversibly aggregate or become proteolytically digested and thus lose its function [1]. To gain more insight into folding kinetics, it is desirable to probe the protein folding transition state with ϕ-value analysis using a series of conservative deletion mutants. The measurement of equilibrium and kinetic constants of these proteins allows a ϕ-value to be calculated with solely folding kinetic data or solely unfolding kinetic data (Eq 1).

$$\phi =\;1-\left(\frac{-RTln\left(\frac{k_{u}^{WT}}{k_{u}^{MUT}}\right)}{\Delta \Delta G_{u}^{o}}\right)$$ (1)

where $k_{u}^{WT}$ and $k_{u}^{MUT}$ are the unfolding kinetic constants of the wild type and mutant respectively, calculated from kinetic experiments and $\Delta \Delta G_{u}^{o}$ is the difference (WT—mutant) in their free energies of unfolding, calculated from equilibrium experiments. The comparison of ϕ_-_values for different mutants can determine the extent each mutation site is fully folded (ϕ-value = 1) or fully unfolded (ϕ-value = 0) in the transition state, and therefore probes the features of the transition state [2]. These data are laborious to collect using traditional methods and therefore such studies have been limited to a few cases.

To obtain these data, the spectroscopic properties of proteins, such as their absorbance, circular dichroism or fluorescence, are measured to assess changes in conformation during unfolding. In a protein unfolding kinetic experiment, these properties are monitored over time after the addition of concentrated denaturant stock, which is usually either guanidinium chloride (referred to as guanidine) or urea to induce unfolding. The final concentration of the denaturant determines how fast the protein will unfold and as such the experiment is repeated several times at different denaturant concentrations as part of a Chevron plot analysis [3]. The kinetic constants measured at each denaturant concentration are then used to determine the protein unfolding kinetic constant in water, which is used in the numerator of Eq 1.

The simplest way to record this data is by the manual addition of denaturant to protein monitored by a spectrometer although this lacks the advantages of automation and does not enable measurement of fast protein folding kinetics. For faster kinetics a stopped flow device connected to a spectrometer is generally used. For the unfolding version of this experiment, the stopped flow device has a syringe filled with protein solution containing denaturant and another syringe with buffer only. These are mixed together in a defined ratio so as to reach the desired final concentration of denaturant and spectroscopic changes are monitored over time. This approach is essential for measuring unfolding kinetic constants that have milli-second half-lives, although many protein unfolding rates are much slower than this with much longer half-lives (seconds to minutes). Furthermore, a full set of kinetic experiments [3] requires at least 20 different concentrations of denaturant [4], which can be quite labor intensive. This method also wastes significant amounts of the protein reagent in syringe/tubing dead spaces. Alternatives to the traditional stopped flow and manual addition methods have been reported that include measuring unfolding rates by pulse proteolysis [5,6] or traditional proteolysis [7,8], and a 96-well plate method using a quantitative real time PCR machine (qRT-PCR) and extrinsic fluorescence dyes [9]. These are introduced below.

The original equilibrium pulse proteolysis method required preparation of protein samples with a range of denaturant concentrations. A brief exposure (or pulse) of a stable protease (such as thermolysin) is used to selectively degrade the unfolded protein fraction in each sample, enabling the folded fraction that remains after the pulse to be measured. Protease-exposed samples are analyzed using SDS-PAGE to quantitate the amount of remaining intact folded protein [5], avoiding the need for a spectrometer. In the kinetic version of this approach, after the addition of a given amount of denaturant, aliquots are taken over time and immediately subjected to a protease pulse and quenched. The amount of folded protein that remains at each time point is characterized by SDS-PAGE and the data fit to an exponential decay function for determination of the rate constant [6]. The pulse proteolysis kinetic approach has the advantage of needing the least equipment and sample and is accessible to most labs since the equipment is common, however, it is quite labor intensive. For each denaturant condition at least one SDS-PAGE experiment is required, necessitating a minimum of 10–20 gels to interrogate the unfolding conditions. Furthermore, this technique requires that the protease only digest the unfolded state, and that the protein unfolds sufficiently slow that the process can be monitored on the minute or greater timescale.

A second reported alternative approach to stopped flow is a modification of the equilibrium differential scanning fluorimetry technique [10], where initial unfolding rates of lysozyme and hexokinase are monitored at different denaturant concentrations in a 96 well plate. The method uses an extrinsic fluorescent dye (Sypro Orange) that must bind quickly and exclusively to the unfolded state and change its emission intensity as the protein unfolds [9]. This approach requires a qRT-PCR machine or plate reader, which is commonly available in life science labs. Furthermore, like the pulse proteolysis method, it is limited to proteins that unfold sufficiently slow that the process can be monitored after the manual addition of denaturant, as the entire plate is measured for each time point.

The third reported approach involves the quantification of intact protein (using analytical chromatography or SDS-PAGE) as the unfolded protein is directly digested by protease [7,8]. In this approach, the kinetic constants for proteolysis are measured at different protease concentrations and fit to a Michaelis-Menten-like equation to yield the unfolding rate of the protein. A requirement is that the rate-limiting step is protein unfolding, and not the intrinsic rate of proteolysis (the EX 1 condition) [11]. The advantage of the approach is that at the concentrations of reagents typically used for common proteases the observed proteolysis rate is much slower than the unfolding rate, and thus measurable without a stopped flow device. This allows the study of proteins with faster unfolding rates compared to the previous two approaches. However, like the pulse proteolysis approach, this method requires absolute protease selectivity towards the unfolded state. It is also labor intensive.

We adapted the stopped flow and proteolysis approaches to measure protein folding kinetics in a plate reader that measures intrinsic tryptophan fluorescence and is equipped with injection syringes. Using this system we developed methods that allow for direct measurement of the unfolding rate of proteins. To benchmark our assay we chose to measure the protein unfolding kinetics of the well-characterized SH3 domain from yeast protein Abp1 [12–14] referred to here as AbpSH3^8–10^ including some of its stabilizing mutants [15]. We also measured the unfolding rates of a AbpSH3-peptide hybrid with a long linker (17 residues) called Hybrid Long Linker (HLL) and tested for inter-molecular peptide binding. Furthermore, we improved the equilibrium stability assay in the plate reader first reported by Dalby and colleagues [16] to obtain an indirect measurement of the folding rate. We use these methods to shed insight into the structure and folding of our domain-peptide hybrid and discuss implications for future high throughput protein unfolding kinetic investigations.

## Materials and Methods

### Materials

The details of the preparation of the construct for Hybrid Long Linker (HLL) were previously reported [17]. The AbpSH3 mutants were obtained from Alan Davidson’s lab, Toronto, Canada. All experiments in a plate reader used a POLARstar Omega machine (BMG labtech, NC, USA) with a Hellma 96 well quartz plate.

### Protein preparation

All proteins were expressed and purified and concentrations determined as previously described [15,17]. Proteins were assayed in 10 mM Tris, 100 mM NaCl, pH 8.0 except SAXS and limited proteolysis assay as indicated below. The pH of the reaction buffer was verified by measurement. Guanidinium chloride (referred to as guanidine) solutions were prepared in reaction buffer. For the kinetic assay, 8 M guanidine was used and for the equilibrium assay, denaturant concentrations between 3 and 6 M were used. The concentration of guanidine solutions were measured using their refractive indices as outlined by Pace [18]. For HLL the final protein concentration in the kinetic assay ranged from 2 to 16 μM and for all others it was 2.5 μM. For the equilibrium assay the initial concentration of all proteins started at 10 μM and typically reduced 3 fold by the end of the experiment. All solutions were filtered and degassed before use.

### Kinetic protein unfolding assay

All assays were performed at 30°C. The globally averaged evaporation rate was determined by adding 300 μL of 10 mM Tris, 100 mM NaCl pH 8.0 to all wells, incubating in the plate reader at 30°C and weighing the plate periodically over a period of 1 hour. The volume change per well per second was calculated as 0.00105 μL/s. We found good agreement between this rate and the rate calculated internally from control wells and used the global rate in our calculations. We tested that injections deposited the correct amount of guanidine into the bottom center of the well and that it was reproducible for both small and large volume injections. We found excellent accuracy and reproducibility down to 3 μL with the 500 μL syringe installed. S1 Table was used to set up the kinetic assays with 24 duplicated samples between 7.5 and 0 M guanidine. For all wells, the final volume before evaporation was 100 μL. For each sequential unfolding experiment, 1000 points are measured from the plate bottom, where each point is an average from 12 lamp flashes over 120 ms. Measurements are made with excitation set at 280 nm (using a 275–285 nm band pass filter) and emission set at 330 nm (using a 325–335 nm band pass filter from the bottom). After measuring a baseline for 10 seconds, a given volume of guanidine is injected into the well while recording fluorescence for a further 1 min 50 seconds. This process is repeated for all samples, where the first well to be measured is the one with the smallest volume of protein/buffer in the well. The data from 12 seconds onwards were fit to an exponential decay curve (Eq 2) to yield an unfolding kinetic constant for each well.

$$y=y_{0}+A_{1}\;e^{-xk_{u}}$$ (2)

where *y* is the observed fluorescence signal, *y*_*0*_ is the initial fluorescence signal, *x* is time, A_1_ is the total fluorescence change or amplitude, and k_u_ is the observed unfolding kinetic constant.

The post injection guanidine concentration for each well was calculated using our template (S1 Table) that takes into account the evaporation that occurred before injection. The natural log of the calculated rate constants was plotted against the denaturant concentration. The part of the data that corresponds to protein unfolding (denaturant concentrations greater than the D_50_ value) were fitted to a straight line (Eq 3) which represents the unfolding arm of a Chevron plot. The intercept of this line is the natural log of the unfolding rate at 0 M denaturant, i.e the rate of unfolding in the system buffer with no denaturant, also known as k_u_ (H_2_O). Errors were reported as standard deviations from at least 2 replicates.

$$\text{ln}k_{u}=\text{ln}k_{u}\;\left({\text{H}}_{2}\text{O}\right)+\frac{m_{u}}{RT}\left[denaturant\right]$$ (3)

where m_u_ is the dependence of unfolding kinetic constants on denaturant concentration multiplied by RT (slope of line x RT), R is the ideal gas constant (8.314 J/mol K), and T is temperature in Kelvin.

### Equilibrium protein unfolding assay

All assays were performed at 30°C. The internal evaporation rate of 0.00173 μL/s was used to calculate the well volumes during the assay, which differs from the kinetic assay due to the presence of guanidine in the buffer and the plate being under partial vacuum. The plate reader injector calibration was performed as described for the kinetic assay and the same filters were used. The plate layout and the 0–4 M template in S2 Table were used to run an equilibrium experiment with 24 titration points over 6 hours in which all wells in the plate were measured before the next data point was collected. After each injection of guanidine, the plate was shaken for 20 s at 100 rpm and 5 data points were recorded during the 15-minute equilibration period before the next injection and this process was repeated for all injections. For each dataset, the last 3 measurement points after each injection were averaged and used as the measurement for that injection in subsequent calculations. During the course of the assay, the sample volume in the well is constantly changing due to evaporation or guanidine injection. Volume changes have a small but significant effect (no greater than 10%) on fluorescence measurements that read from the plate bottom. To correct for this effect, 4 control wells were included in which protein was already unfolded in 8 M guanidine and the same denaturant injections were made to these wells. The data from these control wells were used to calculate 24 correction factors to be applied to the rest of the data. Each correction factor at a given injection was calculated by dividing the first control measurement by the control measurement at that injection and this value was averaged for the 4 replicates. The rest of the data at a given injection was multiplied by the corresponding averaged correction factor. For each protein, the resultant volume corrected fluorescence emission data were plotted against the evaporation corrected denaturant concentrations and fit to Eq 4. Errors were reported as standard deviations from at least 3 replicates.

$$Y=\frac{\left(Y_{F}+m_{F}\left[D\right]\right)+\left(Y_{U}+m_{U}\left[D\right]\right)exp^{\left(\frac{m\left(\left[D\right]-D_{50}\right)}{RT}\right)}}{1+\;exp^{\left(\frac{m\left(\left[D\right]-D_{50}\right)}{RT}\right)}}$$ (4)

where Y is the overall fluorescence signal, m is the equilibrium m-value, m and Y with the subscripts F and U refer to the folded and unfolded state baseline slopes and fluorescence intercepts respectively. [D] is denaturant concentration and D_50_ refers to the 50% midpoint denaturant concentration.

### Gel filtration

8 μL samples of HLL (at concentrations of 62.5 μM) were run at 0.3 mL/min on a Superdex 75 5/150 GL equilibrated with 50 mM Tris, 300 mM NaCl pH 8.0. A standard curve (log MW vs. Elution Volume) was prepared using bovine serum albumin, carbonic anhydrase, myoglobin and cytochrome c with molecular weights (MW) of 68, 30, 18.8 and 12.3 kDa respectively. The mass of HLL was estimated from the standard curve (S1 Fig).

### Small angle x-ray scattering (SAXS)

Protein sample for SAXS were extensively dialyzed against 50 mM phosphate, 100 mM NaCl, pH 7.0 and filtered through a 0.22 micron filter immediately preceding SAXS data collection. SAXS data were collected with protein concentrations ranging from 1.5–4.7 mg/mL with a Bruker Nanostar system equipped with a rotating anode (Cu) Turbo X-ray Source and a Vantec-2000 (2048 x 2048 pixel) detector (Bruker AXS). The sample-to-detector distance was set at approximately 67 cm allowing for the detection range: 0.012 Å^-1^ > *q* > 0.383 Å^-1^. Sample and buffer scattering data were each collected for 3–6 hours. The SAXS data sets were averaged and converted to 1D scattering profiles for further analysis. The ATSAS [19] software suite was used to carry out buffer subtraction and process the SAXS data. The radius of gyration (*R*_g_) was determined by using the Guinier approximation in the *q* range, such that *q*_max_ ≤ *R*_g_ ≤ 1.3.

### Limited proteolysis

For all experiments, the final protein concentration was 20 μM in 100 μL 50 mM Tris, 100 mM NaCl, 100 mM CaCl_2_ pH 8.0 and the final concentration of protease ranged from 0.05 μM to 20 μM. First, the appropriate amounts of buffer and enzyme were added to a separate 96 well plate and covered, then the protein was added to a 96 well quartz plate and covered. With a multichannel pipette, the enzyme and buffer mixture in the separate plate was added to the quartz plate immediately before the experiment was run. The plate was covered with parafilm to prevent evaporation in the plate reader during the course of the 12 hours of measurements. The raw fluorescence data (excitation 280 nm, emission 330 nm) for each enzyme concentration were fitted to an exponential decay function to yield an observed kinetic constant (Eq 2).

The kinetic constant data were plotted against enzyme concentration and hyperbolic curves were fitted to Eq 5 to yield the unfolding constant k_u_. The whole $\frac{k_{\text{u}}}{{\text{K}}_{\text{u}}\left(\frac{k_{\text{cat}}}{{\text{K}}_{\text{M}}}\right)}$ term was fixed as one independent variable during the fitting process [20].

$$k_{\text{obs}}=\;\frac{k_{\text{u}}\left[\text{E}\right]}{\frac{k_{\text{u}}}{{\text{K}}_{\text{u}}\left(\frac{k_{\text{cat}}}{{\text{K}}_{\text{M}}}\right)}\;+\;\left[\text{E}\right]}$$ (5)

where k_obs_ is the observed kinetic constant for degradation under EX1 conditions, k_u_ is the unfolding kinetic constant, [E] is the protease concentration, K_u_ is the equilibrium constant for unfolding, k_cat_ is the catalytic rate constant for the protease and K_M_ is the Michaelis constant for the protease.

## Results and Discussion

### Proteins of study

An active research effort in our laboratory requires measurement of the unfolding rate of the AbpSH3-ArkA peptide hybrid called HLL [17]. Further, our ultimate goal is to measure a large number of mutants in a variety of buffer conditions, making the development of a fully automated protein folding kinetic assay highly desirable. The folding kinetics and stability of AbpSH3 and several stabilized mutants are well defined [15] and allow us to conveniently benchmark our method against this previously collected data. The unfolding kinetic constant for the AbpSH3 domain was expected to decrease when the ArkA binding peptide is covalently attached by a flexible linker as observed in other SH3 domain-peptide hybrids [21].

### Kinetic protein unfolding assay

The assay we developed measures protein unfolding by injecting concentrated denaturant into a well and following the change in fluorescence emission over time. The assay relies on a plate reader that makes an injection at the same location as the reading head, such that the contents of the well are continuously measured before, during and after the injection. Thus, we used a BMG POLARstar Omega plate reader that offers injections at the point of measurement, retaining the maximum amount of signal information. Furthermore, we optimized the reading mode by choosing to excite and collect fluorescence emission from below the plate and away from the injection head. This is made possible through the use of a Hellma 96 well quartz plate that provides practically no interference for bottom measurements. Bottom measurements also result in little interference from the changing meniscus position and no risk of injecting denaturant onto the reading head. We injected denaturant at a fast rate (430 μL/sec) with smaller volumes, and slower rates (170 μL/sec) with the bigger volumes, which maximized mixing and minimized splashing between wells. Also, by lowering the number of lamp flashes per measurement, we were able to measure fluorescence emission as often as every 120 ms with no photodegradation across the experiment. This plate reader also has the advantage of using band pass filters for the fluorescence measurements, which gives much more stable readings compared to plate readers with monochromators that can be prone to substantial baseline drift. In agreement with Dalby and colleagues [16], we found 3 μL to be the smallest highly reproducible injection volume using a 500 μL syringe.

The general steps for measuring unfolding kinetics are outlined below:

1. Measure the evaporation rates.
  In a separate experiment, perform a global evaporation rate measurement to calculate evaporation rate from the plate during the assay. To complement this measurement, as part of the assay, include 4 wells with 300 μL reaction buffer and measure their volumes at the end for an internal evaporation rate (S1 Table).
2. Program plate reader method and prepare samples.
  Specify injection volumes and all other general parameters as discussed in methods section. Use S1 Table to prepare 24 duplicated protein samples with guanidine concentrations between 7.5 and 0 M in microcentrifuge tubes. To reduce the number of samples one can choose 8–12 concentrations (no duplicates) to span the range between the protein’s D_50_ and the maximum denaturant concentration.
3. Prime syringe.
  Slowly prime one injector pump with guanidine solution in reaction buffer, ensure there are no air bubbles and place injector needle in reading head. As an option, test injections are accurate in a spare plate.
4. Fill border wells.
  To avoid greater evaporation that occurs from the wells on the edges of the plate, fill the border wells with 300 uL of reaction buffer, and use only the interior wells for the experiment. Fill any unused wells with buffer as well (S1 Table).
5. Fill sample wells.
  Load protein samples into the plate and check all samples have similar fluorescence emission values. Optimize the gain level on a well that contains at least 50 μL of protein so that the fluorescence intensity is ~50% of the maximum value the instrument can accurately measure.
6. Run the experiment.
  Monitor the first few injections to check that the system is behaving as expected.
7. Make manual measurement at end of the experiment.
  Measure the volumes of the internal evaporation control wells (step 2) to calculate the final guanidine concentrations.

**Fig 1:**
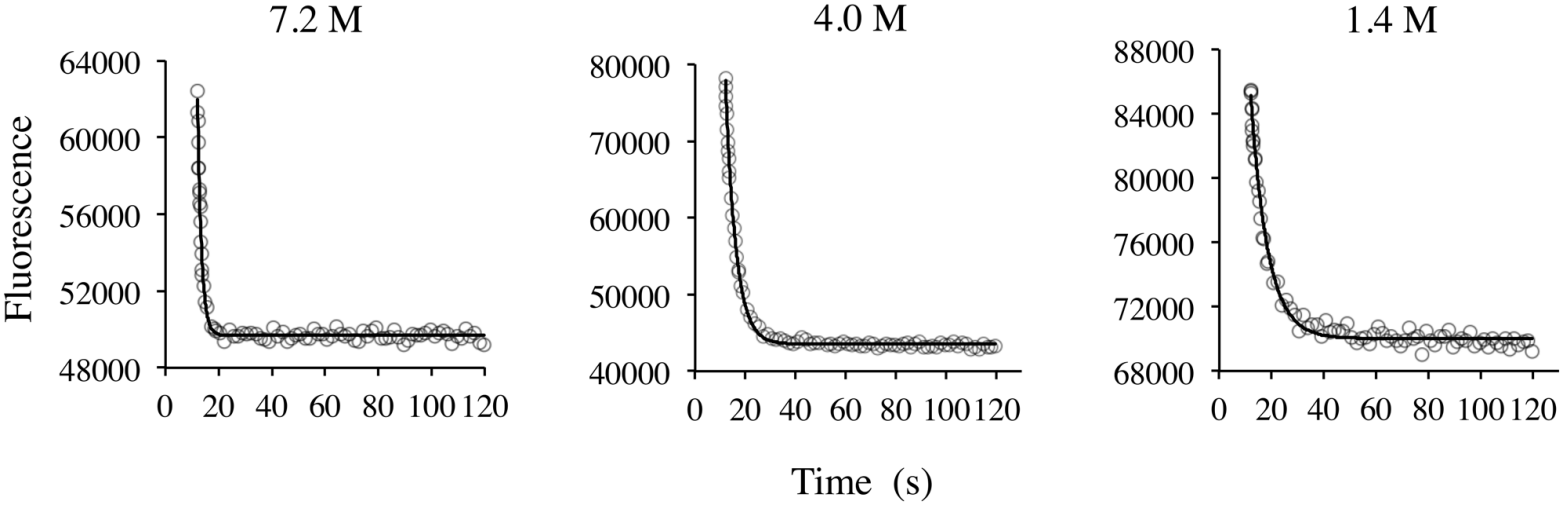
Representative kinetic traces generated from our plate reader method for AbpSH3 WT. The final guanidine concentration is at the top of each graph. Excitation is at 280 nm and emission is at 330 nm. The black line is fit to an exponential decay (Eq 2). Guanidine injection starts at 10 s.

A typical (partial) set of processed data from our plate reader unfolding experiments can be seen in Fig 1 and S2 Fig that present unfolding curves are generated at different final concentrations of guanidine and data (after 12 seconds) that are fitted to Eq 2.

**Fig 2:**
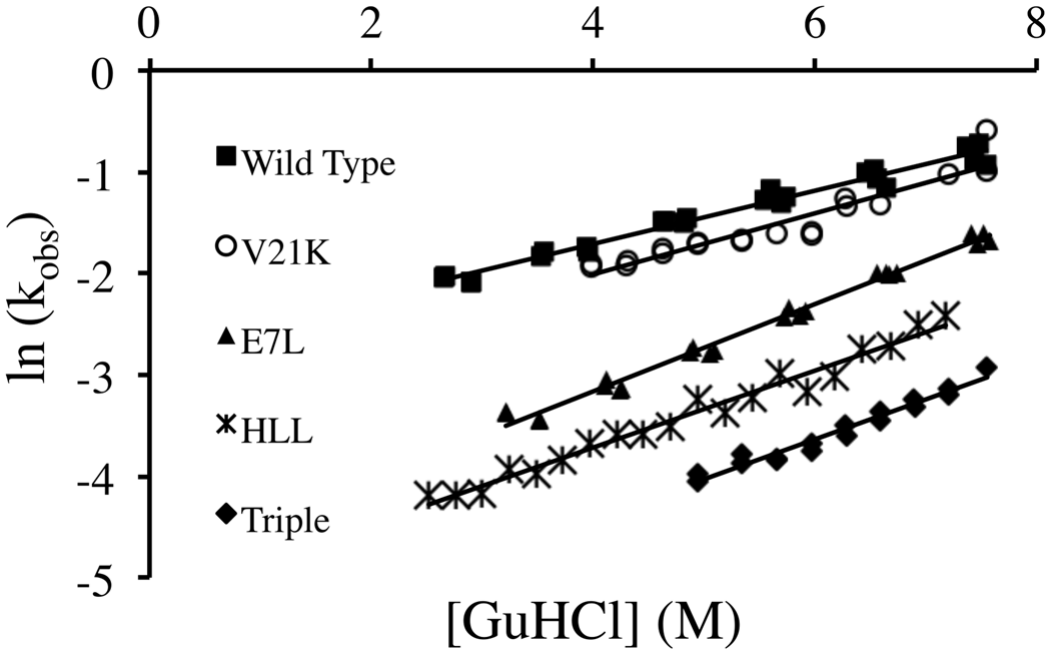
Representative Chevron plot unfolding arms. For each protein, unfolding kinetic constants (k_u_) were determined with varying final concentrations of guanidine. From the fitted line for each dataset, the y-intercept is ln (k_u_ (H_2_O)) and the slope is $\frac{m_{u}}{RT}$ as indicated in Eq 3.

As can be seen from Fig 2 and S3 Fig, a well-fitted, reproducible unfolding arm is obtained for AbpSH3 and related proteins. The data points in each Chevron plot become more scattered at the higher concentrations of guanidine, suggesting that rate constants higher than 0.9 s^-1^ (which corresponds to a half life of 0.77 s) are close to the detection limit for this method. As can be seen in Table 1, for AbpSH3, the intercept from this data gives a k_u_ (H_2_O) of 0.071 s^-1^ and a m_u_ of 0.92 kJ/mol M, which are close to literature values of 0.066 s^-1^ and 0.83 kJ/mol M respectively [22]. It is noted that whether the protein and denaturant solutions are mixed in a ratio of 2:1 or 1:10, the rapid mixing in the well still provides essentially equivalent kinetic information for the protein (S1 File)

**Table 1:** Summary of kinetic unfolding data collected using the plate reader method.

	k_u_ experimental (s^-1^) X 10^3^	k_u_ literature (s^-1^) X 10^3^	m_u_ experimental (kJ mol^-1^ M^-1^)	m_u_ literature (kJ mol^-1^ M^-1^)
**AbpSH3 WT**	71.0 ± 5.4	65.8 ± 5.3	0.92 ± 0.06	0.83 ± 0.04
**AbpSH3 E7L**	7.2 ± 0.5	4.8 ± 1.6	1.09 ± 0.01	0.96 ± 0.10
**AbpSH3 V21K**	48.5 ± 10.5	44.8 ± 4.3*	0.69 ± 0.05	0.56 ± 0.03*
**AbpSH3 Triple**	2.9 ± 0.6	4.7 ± 3.6	1.01 ± 0.01	0.70 ± 0.20
**HLL**	4.2 ± 0.8	n/a	1.07 ± 0.01	n/a

* refers experiments using urea instead of guanidine as the denaturant. The HLL experiment consisted of an average of 4 experiments using different end protein concentrations (2 to 16 μM). All other experiments were performed at least twice. The n/a indicates that there is no literature value available for comparison.

### Further validation and data from SH3 domain-peptide hybrid

In our subsequent kinetics experiments the guanidine stock concentration was fixed to approximately 8 M. The described method was tested on three previously characterized AbpSH3 mutants, E7L and V21K are single mutants and “Triple” contains E7L, V21K and N23G mutations. A recently expressed and purified AbpSH3 domain-peptide hybrid, HLL, was also tested [17].

Table 1 provides the unfolding kinetic constants and m-values for the above proteins as well as their literature values [15,22]. The unfolding rates and m-values agree extremely well with known literature values with excellent standard deviations, making this assay comparable to stopped-flow approaches for these proteins.

As expected, the hybrid shows a reduction in its unfolding rate as the binding peptide (lacking tryptophans and therefore providing no contribution to the fluorescence signal) increases the stability of the SH3 domain native state through mass action. Experiments for this hybrid was repeated at four different protein concentrations ranging from 2 to 16 μM and the small unfolding rate changes were within the error limits of our experiment (Table 1 provides average). Furthermore HLL fits very well to a classic 2-state unfolding process, which suggests no unfolding intermediate exists, such as an isolated folded domain with no bound peptide. These data and results from previous studies [21,23,24] suggest that the domain in HLL binds peptide most likely via an intra-molecular interaction.

**Fig 3:**
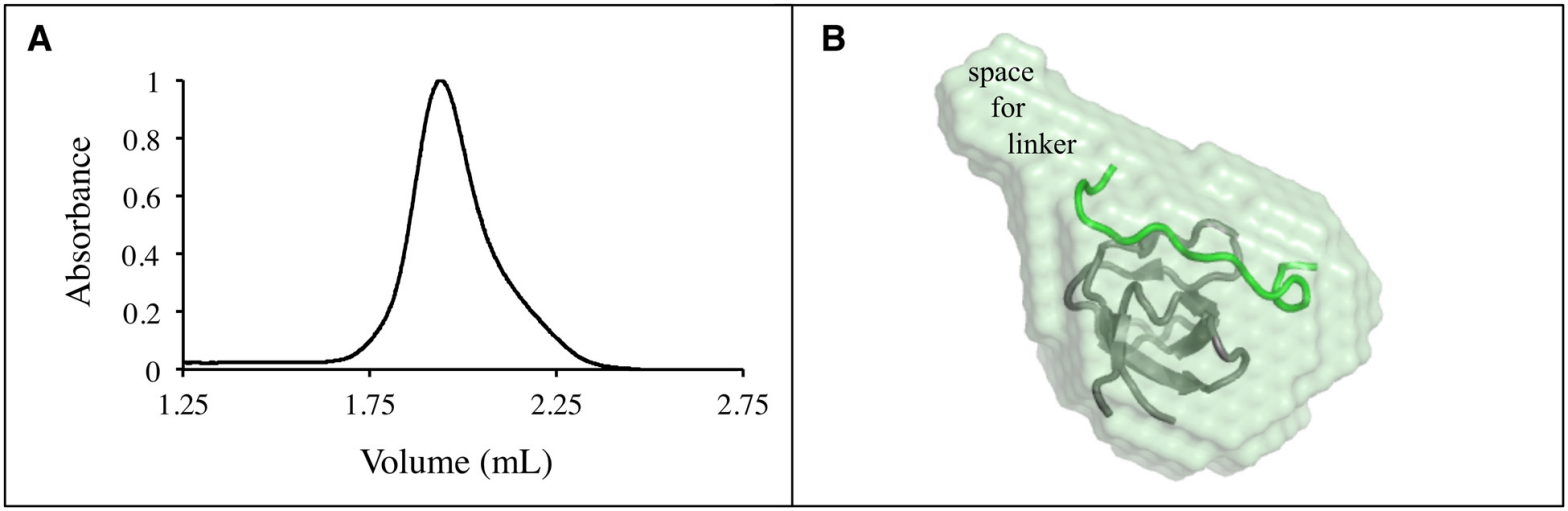
SAXS and gel filtration chromatography of HLL. (A) Gel filtration chromatogram of the HLL sample. Absorbances are reported at 205 nm and expressed as a fraction of the maximum change. (B) Molecular envelope is calculated for HLL. Inside the envelope is a cartoon representation of the AbpSH3-ArkA complex (pdb code 2rpn) and a label indicating an area to fit the linker that connects the C-terminus of the domain (grey) to the N-terminus of the peptide (green).

To confirm that the peptide binding to the domain in HLL is via an intra-molecular interaction and check for the possibility of oligomerization, we performed small angle x-ray scattering (SAXS) and gel filtration chromatography of HLL (Fig 3). The calculated SAXS molecular envelope agrees well with the known AbpSH3-ArkA complex structure with expected additional space for the flexible 17 amino acid linker. The theoretical MW from the SAXS data [25] is estimated as 12.7 kDa (radius of gyration of 17.3 Å), while by gel filtration (standard curve shown in S1 Fig) it is estimated as 13.3 kDa. Both measurements provides values close to the expected MW of 12.4 kDa.

Thus our kinetic data combined with additional biophysical data indicates that HLL is a compact intra-molecular complex that is substantially more kinetically stable than the domain alone.

### Equilibrium protein unfolding assay

To obtain equilibrium stability measurements for these proteins, we used plate reader based isothermal equilibrium denaturation experiments in a plate reader. This assay involves serial additions of guanidine to a protein in aqueous buffer first described for plate readers by Dalby and colleagues [16]. We modified the original method by shortening the interval time between injections to 15 minutes, detecting from the bottom of the plate and using both injection pumps. Each injection pump provides different denaturant stocks for better denaturant concentration control during the titration. Furthermore we adjusted for signal fluctuations caused by dilution and evaporation volume changes during the experiment. The general equilibrium unfolding assay is outlined below:

1. Measure the evaporation rate.
  The longer assay duration will lead to condensed water build up inside the reader, which is minimized by applying a partial vacuum. As such, another global evaporation rate measurement is recommended using the same method as the kinetic assay.
2. Program plate reader method.
  Use S2 Table to determine a suitable plate reader method that will yield good pre and post unfolding baselines and sufficient points through the transition region (a minimum of 24 points in total). Specify all other general parameters as found in methods section.
3. Prepare samples.
  Use S2 Table to prepare sufficient protein in reaction buffer to allow for 50 μL of 10 μM protein per well, in triplicate. Also prepare 4 wells of a known protein in denaturant solution (usually 8 M guanidine in reaction buffer) to act as a control for volume changes (step 7).
4. Prime syringes and fill border wells
  Slowly prime both injector pumps with the appropriate guanidine solutions (S2 Table) and check that they inject correctly as in previous kinetic assay.
5. Fill border/sample wells and run the experiment.
  Fill border wells (and any other unused wells), fill sample wells, and determine gain value as in previous kinetic assay. Turn on vacuum and run the experiment.
6. Make manual measurement at end of experiment.
  After the experiment has finished, carefully measure the volumes of 4 randomly chosen samples to calculate an internal evaporation rate.
7. Process data before fitting.
  Adjust the experimental fluorescence data by multiplying by the volume correction factors calculated from the titrations of protein that are already unfolded (step 3). Use the internal evaporation rate (step 6) and S2 Table to calculate the final guanidine concentrations.

**Fig 4:**
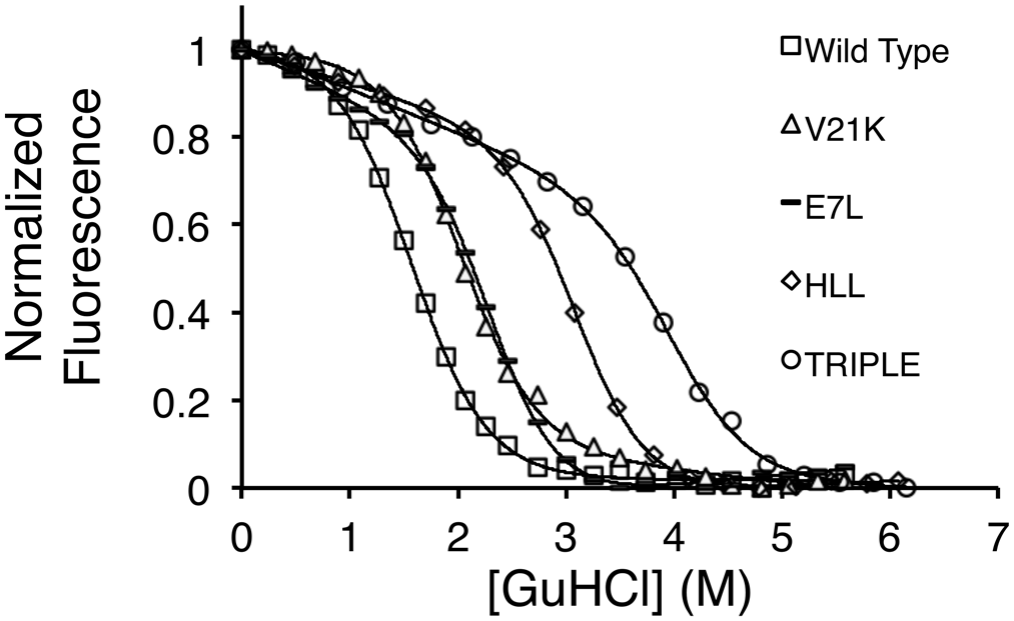
Representative guanidine denaturation curves of proteins. AbpSH3 WT (open square), V21K (open triangle), E7L (line), Triple (open circle) and HLL (open diamond) denaturation monitored by tryptophan fluorescence emission at 330 nm. Fluorescence values are expressed as a fraction of the total change. The lines joining the points in each graph are theoretical fits to the data based on Eq 4 and the resultant thermodynamic parameters are shown in Table 2.

Typical data sets with fitted curves can be seen in Fig 4 and S4 Fig and indicate good quality data and fits are provided through this approach.

As can be seen in Table 2, we obtained reproducible stability data that corresponded well with known literature values. In principle, it is possible to calculate an indirect folding rate using the unfolding rate and the equilibrium constant. We observed the folding constants obtained by this approach were on the same order of magnitude as literature values (S2 File).

**Table 2:** Summary of equilibrium data collected using the plate reader method. All proteins are WT or mutants of AbpSH3 except HLL. The literature m_eq_ and D_50_ values for V21K were omitted as they were only recorded using urea and not guanidine. “Lit” indicates the literature value and “exp” the experimental value measured in this study, “n/a” indicates that there is no literature value available for comparison. D_50_ units are M, m_eq_ units are kJ mol^-1^ M^-1^, and ΔG_u_ units are kJ mol^-1^.

	m_eq_ exp.	m_eq_ lit.	D_50_ exp.	D_50_ lit.	ΔG_u_ exp.	ΔG_u_ lit.
**WT**	7.87 ± 0.07	6.86	1.64 ± 0.02	1.88	12.92 ± 0.04	12.89
**E7L**	7.46 ± 0.66	7.03	2.32 ± 0.07	2.57	17.31 ± 1.51	18.03
**V21K**	7.20 ± 0.44	n/a	2.02 ± 0.01	n/a	14.55 ± 0.93	14.48
**Triple**	6.49 ± 0.24	6.86	4.05 ± 0.01	3.92	26.29 ± 0.94	26.90
**HLL**	7.33 ± 0.80	n/a	3.12 ± 0.02	n/a	22.89 ± 2.49	n/a

In an effort to maximize efficiency, we combined equilibrium and kinetic methods into one experiment, where all relevant guanidine concentrations needed for both assays were measured in the kinetic assay. The final time points from each decay curve were used to generate an equilibrium denaturation curve from this data. This simultaneous acquisition method gave adequate kinetic data and was an excellent initial indicator of the overall protein stability. However, the equilibrium data was more scattered and the first equilibrium assay approach described above is the more reliable method.

### Probing faster unfolding rates using a plate reader based limited proteolysis assay

Unfolding or folding kinetic constants greater than 0.01 s^-1^ are not be amenable to our technique as described here. The dead time of the experiments is approximately 2 seconds, therefore almost all protein would have unfolded (or folded) in that time. However, modifying our high-throughput method with limited proteolysis can circumvent this problem. Literature examples indicate proteolysis can monitor global unfolding under native conditions [5,7,8,11]. As such the rate of proteolysis (which is substantially slower) can be used to determine the protein unfolding rate. As a proof of principle we decided to test this method using AbpSH3 and the AbpSH3-peptide hybrid, with trypsin and thermolysin proteases.

**Fig 5:**
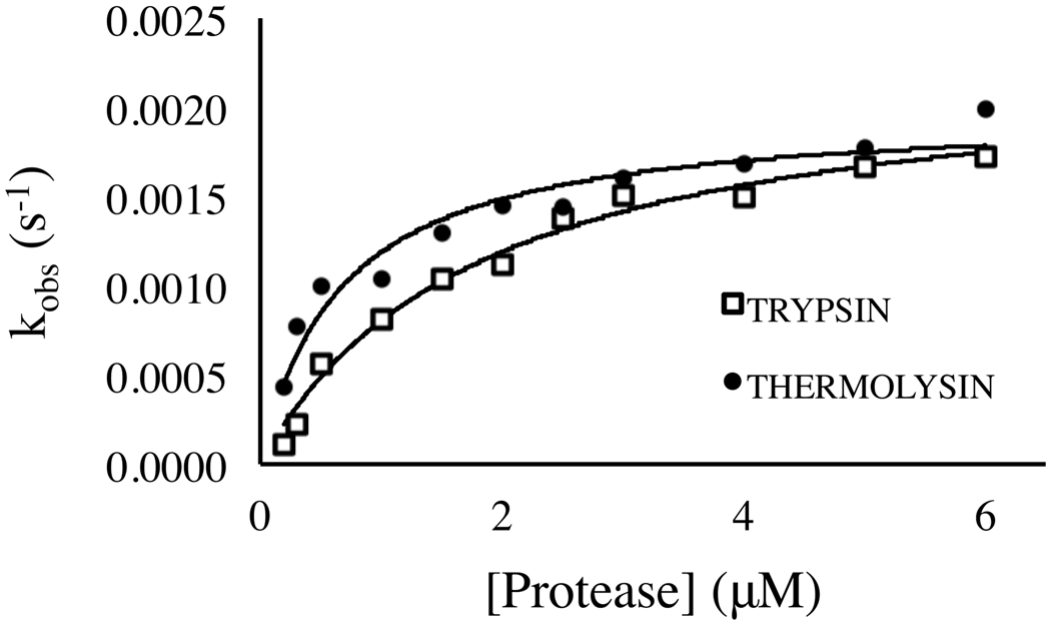
Unfolding kinetic constant derived from limited proteolysis of HLL. A range of concentrations of trypsin (A) and thermolysin (B) are used and degradation is followed by tryptophan fluorescence emission at 330 nm. The fits are to Eq 5 as indicated in the methods.

In our assay, each covered-well contained 100 μL of 20 μM protein with differing concentrations of protease ranging from 0 to 20 μM. Intrinsic tryptophan fluorescence emission of the protein was monitored over time in the same way as the above described assays. Similar to protein unfolding, protein degradation exposes all amino acids to the solvent leading to a fluorescence change amenable to fitting with Eq 2 and with apparent first order kinetics (despite the possibility of multiple cleavage events) [26]. The kinetic constants are plotted against protease concentration to yield either an unfolding kinetic constant (under the EX1 condition) or a native state equilibrium constant for the conversion to the protease susceptible state (under the EX2 condition). Trypsin and thermolysin digestion of both AbpSH3 and HLL indicates AbpSH3 is proteolized under the EX2 condition. This conclusion was drawn from the observed linear relationship between the apparent kinetic constant vs. concentration with both proteases (data not shown). Further study is required to reveal the protease susceptible equilibrium constant. For HLL, protease degradation did not occur through fast native state fluctuations but through the slower conversion to the unfolded state under the EX1 condition. This is deducted from the hyperbolic nature of the plot (Fig 5), which upon fitting with Eq 5, yields unfolding constants for the trypsin and thermolysin experiments of 0.0023 s^-1^ and 0.0022 s^-1^ respectively (compared to 0.0042 s^-1^ from Table 1). By this complementary method, fast unfolding proteins may still provide reliable kinetic measurements using high throughput plate reader assays.

## Conclusions

Currently, about two thirds of the proteins in the protein folding kinetics database [27] have protein unfolding rates that in principle could have been measured using the kinetic unfolding assay described here (k_u_ smaller than 0.8 s^-1^). The dead-time for the assay is 2 seconds compared to >5 seconds with the qRT-PCR method which relies on manual addition of denaturant, can only record data points every 5 seconds (as opposed to every 120 ms in our assay) and relies on an extrinsic fluorescent probe (an additional limitation). Furthermore, the pulse proteolysis method also suffers from longer dead-times, is restricted by the proteolysis mechanism for each protein under study and is not automated. Each unfolding curve in this assay only requires 100 μL of 2.5 μM protein, with no need for extra sample to prime lines and fill dead volumes. With this assay we quickly established that the basis of stabilization for the hybrid is predominantly due to a much slower unfolding rate (approximately 20 fold slower than AbpSH3) as we predicted. Future mutational studies on this system promise to reveal the importance of linker length and composition to the hybrid’s folding behavior [21,23,24,28,29].

A useful application of this assay would be to screen large libraries of mutants for individuals with increased kinetic stability by choosing just one final denaturant concentration for every mutant. Alternatively, the equilibrium assay could be run first on the library and with the top hits, choose 4 final denaturant concentrations for each mutant to get complementary kinetic data. In addition, our kinetic screen will also allow for a complimentary method for screening drug libraries, as drug binding can decrease protein unfolding kinetics [6]. Similarly, this assay could be used to rapidly find stabilizing additives and optimal buffer conditions for long term protein storage. Furthermore, the limited proteolysis assay will be an excellent complement to chemical denaturation, extending automation to faster unfolding proteins and avoiding chemical denaturants.

Site directed saturation mutagenesis and synthetic gene technologies now make the production of mutant libraries quick and economical. With mutants in hand, in theory, within a 96 well plate, one could interleave 7 different mutants, each with 7 points in their unfolding arm. As such one could obtain 7 accurate unfolding kinetic constants in as little as 2 hours instrument time and thus easily repeat this experiment several times in one day. In combination with our highly accurate equilibrium assay, one would be able to calculate ϕ_-_values for these mutants. This would greatly speed up ϕ-value analysis and allow the nature of a folding transition state to be more extensively characterized. Whereas previous protein folding studies have typically been limited to individual proteins, the assays described in this study now open the way for high-throughput kinetic characterizations.

## Supporting Information

S1 Fig Standard curve for gel filtration molecular weight determination. The data was fitted to a straight line and the measured elution volume of 1.94 mL was converted into a MW of 13.3 kDa for HLL using the equation indicated.
(PDF)

S2 Fig Set of kinetic traces generated from our plate reader method for AbpSH3 WT. The final guanidine concentration is at the top of each graph. Excitation is at 280 nm and emission is at 330 nm. The white line is fit to an exponential decay (Eq 2). Guanidine injection starts at 10 s and data are fit at 12 s.
(PDF)

S3 Fig Duplicate chevron plot unfolding arms of mutant proteins. (PDF)

S4 Fig Guanidine denaturation curves with standard deviations included. Data comes from triplicate samples.
(PDF)

S1 File Comparison of unfolding kinetic constants when the same final concentration of guanidine is reached using different protein:denaturant volume ratios. In almost all cases, the unfolding kinetic constants for AbpSH3 are very similar regardless of stock denaturant concentration used in the syringe.
(PDF)

S2 File Indirect folding calculations from k_u_, m_eq_ and D_50_ values give estimates of the folding rates. (PDF)

S1 Table Templates for kinetic assay. There is a sample set up tab and a plate layout tab.
(XLSX)

S2 Table Templates for equilibrium assay. There is a sample set up tab and a plate layout tab. In addition there is a choice of 3 injection protocols; the 0 to 4.0 M protocol (proteins with D_50_ values around 2), the 0 to 4.8 M protocol (D_50_ values around 3) and the 1 to 5.4 M for the most stable proteins.
(XLSX)
