# Supplementary figures and images for: Development and Application of a High Throughput Protein Unfolding Kinetic Assay
Source: PLoS One. 2016 Jan 8;11(1):e0146232. doi: 10.1371/journal.pone.0146232 (PMC4706425; doi:10.1371/journal.pone.0146232)

## Supplementary Figure 1

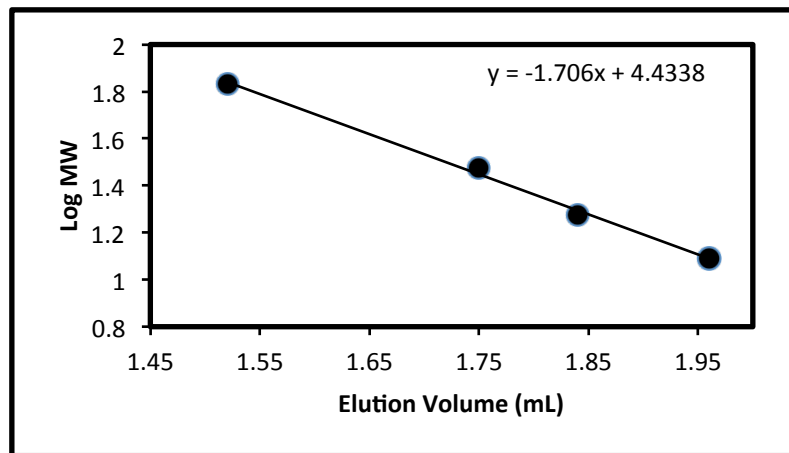

Supplement: S1 Fig — The data was fitted to a straight line and the measured elution volume of 1.94 mL was converted into a MW of 13.3 kDa for HLL using the equation indicated. (PDF) [file pone.0146232.s001.pdf]

Supplementary Figure 2

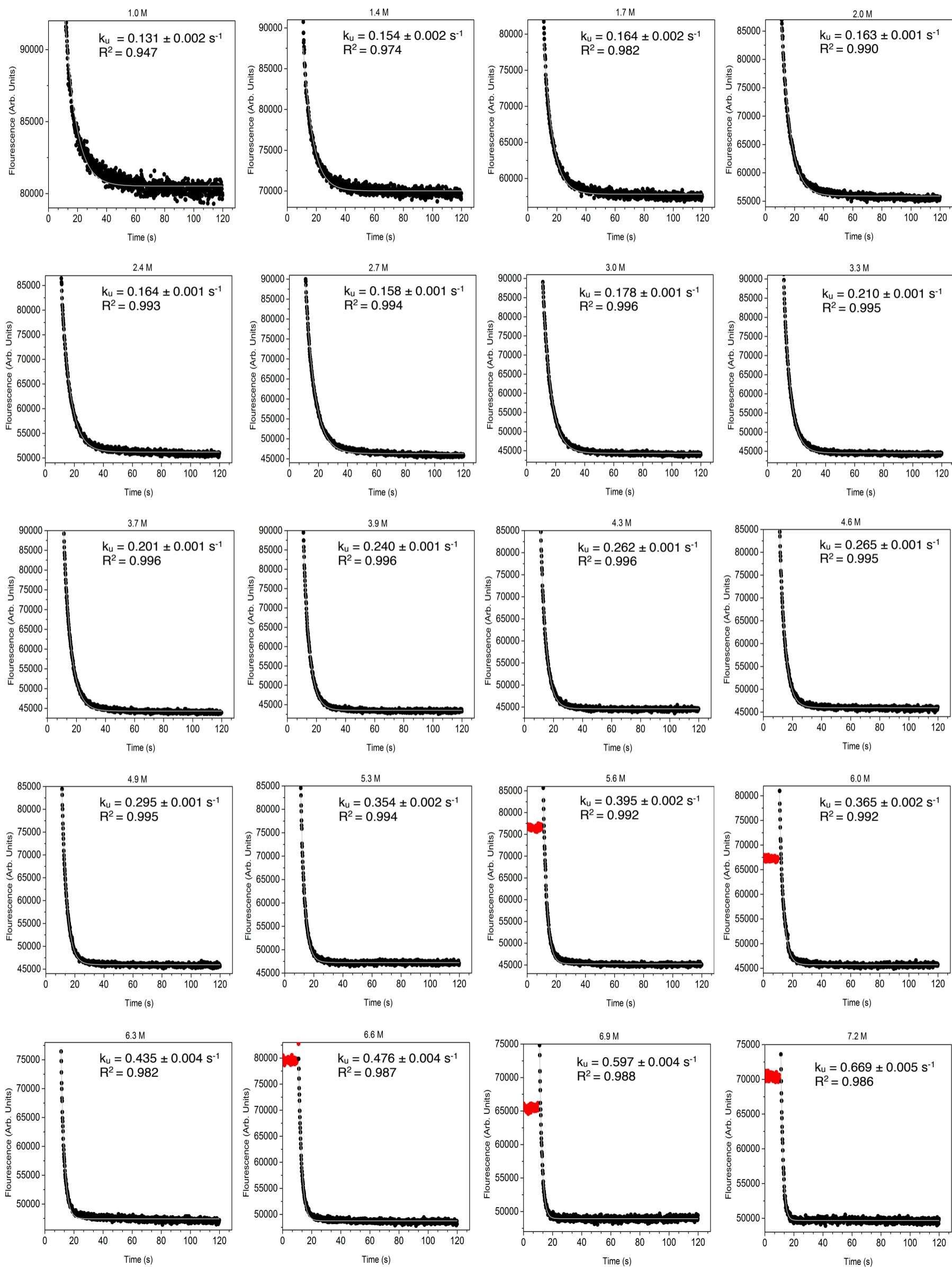

Supplement: S2 Fig — The final guanidine concentration is at the top of each graph. Excitation is at 280 nm and emission is at 330 nm. The white line is fit to an exponential decay (Eq 2). Guanidine injection starts at 10 s and data are fit at 12 s. (PDF) [file pone.0146232.s002.pdf]

## Supplementary Figure 3

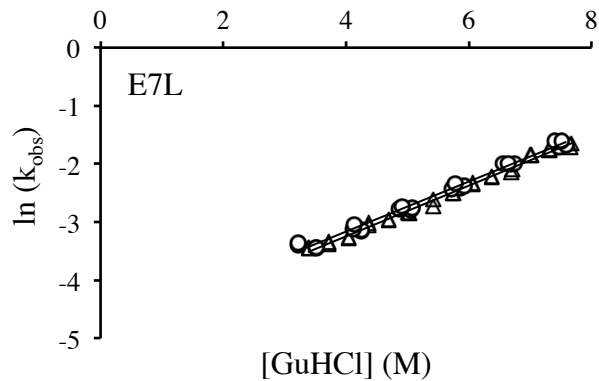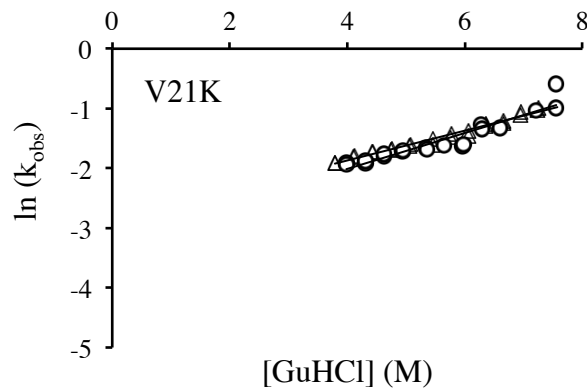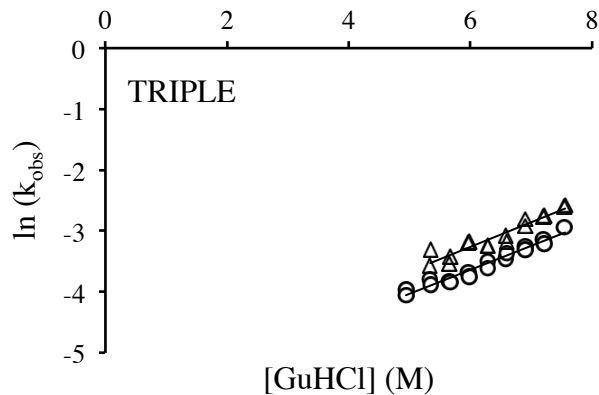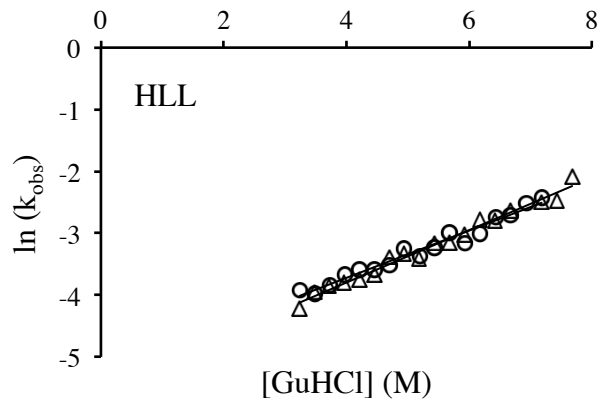

Supplement: S3 Fig — (PDF) [file pone.0146232.s003.pdf]

## Supplementary Figure 4

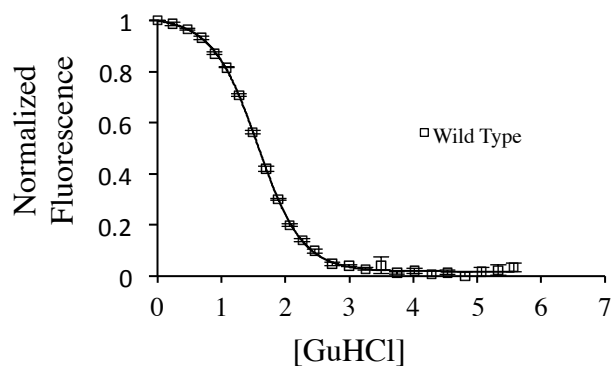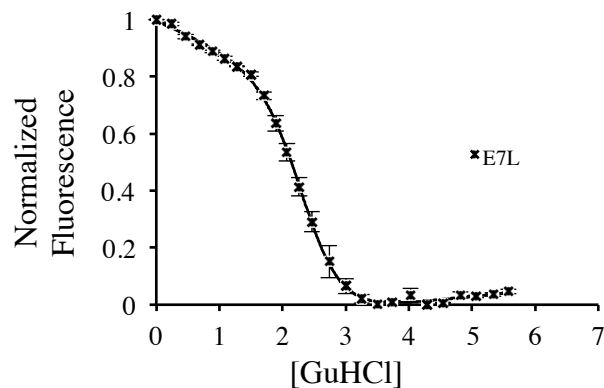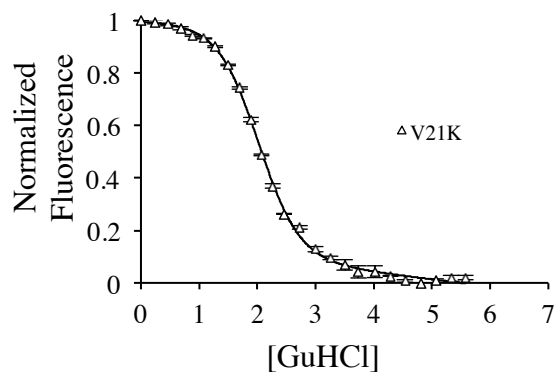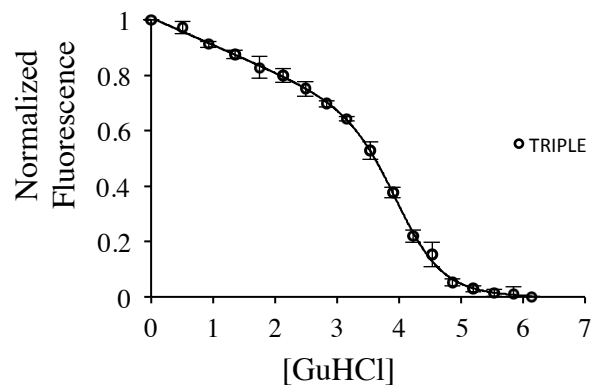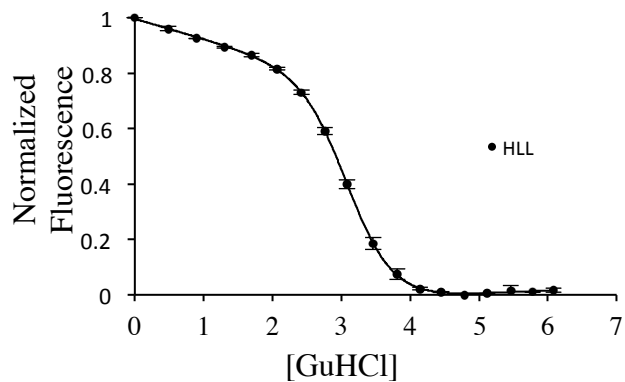

Supplement: S4 Fig — Data comes from triplicate samples. (PDF) [file pone.0146232.s004.pdf]
