# Supplementary material for: Development and Application of a High Throughput Protein Unfolding Kinetic Assay
Source: PLoS One. 2016 Jan 8;11(1):e0146232. doi: 10.1371/journal.pone.0146232 (PMC4706425; doi:10.1371/journal.pone.0146232)
Supplement: S1 File — In almost all cases, the unfolding kinetic constants for AbpSH3 are very similar regardless of stock denaturant concentration used in the syringe. (PDF) [file pone.0146232.s005.pdf]

Supplementary File 1

Effect of different protein:denaturant volume ratios

In a typical stopped flow experiment, the ratio of protein solution to denaturant solution that is mixed together is usually 1:10 and this ratio is fixed for all experiments that measure unfolding [1]. In our kinetic unfolding method to achieve the various different denaturant conditions we change this ratio for every different condition measured. To measure the mixing efficiencies at different ratios, we compared kinetic datasets that used different denaturant solutions in the injection syringe. As can be seen in Fig. A, the y-intercept; ln (k_u (H_2O)) and the slope; $\frac{m_u}{RT}$ for AbpSH3 were very similar regardless of the guanidine stock used. This data reveals unfolding kinetic constants of 0.77, 0.069, 0.072 and 0.064 s^{-1} for 6.02, 6.51, 6.95 and 7.49 M guanidine stocks in the syringe respectively, with an average value of 0.071 ± 0.005 s^{-1}, which compares to 0.066 s^{-1} as reported by Davidson and colleagues [2].

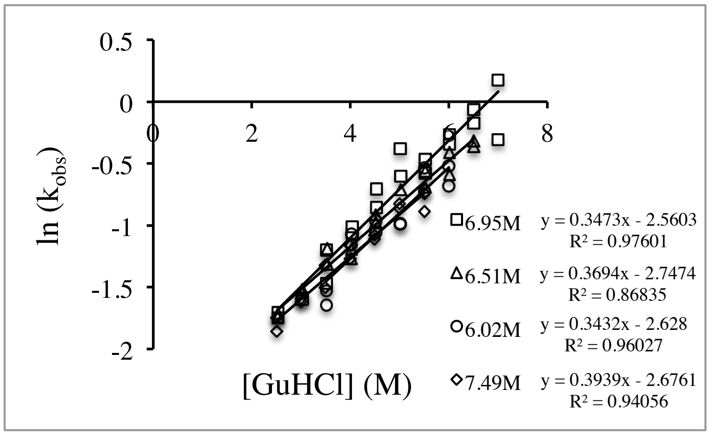

Fig. A Chevron plot unfolding arms for AbpSH3 WT from experiments that used different guanidine concentrations in the injection syringe. From the fitted line for each dataset, the y-intercept is ln(k_u (H_2O)) and the slope is $\frac{m_u}{RT}$.

Most of the unfolding kinetic constants at comparable final denaturant concentrations were very similar as well (Fig. B)

Supplementary File 1

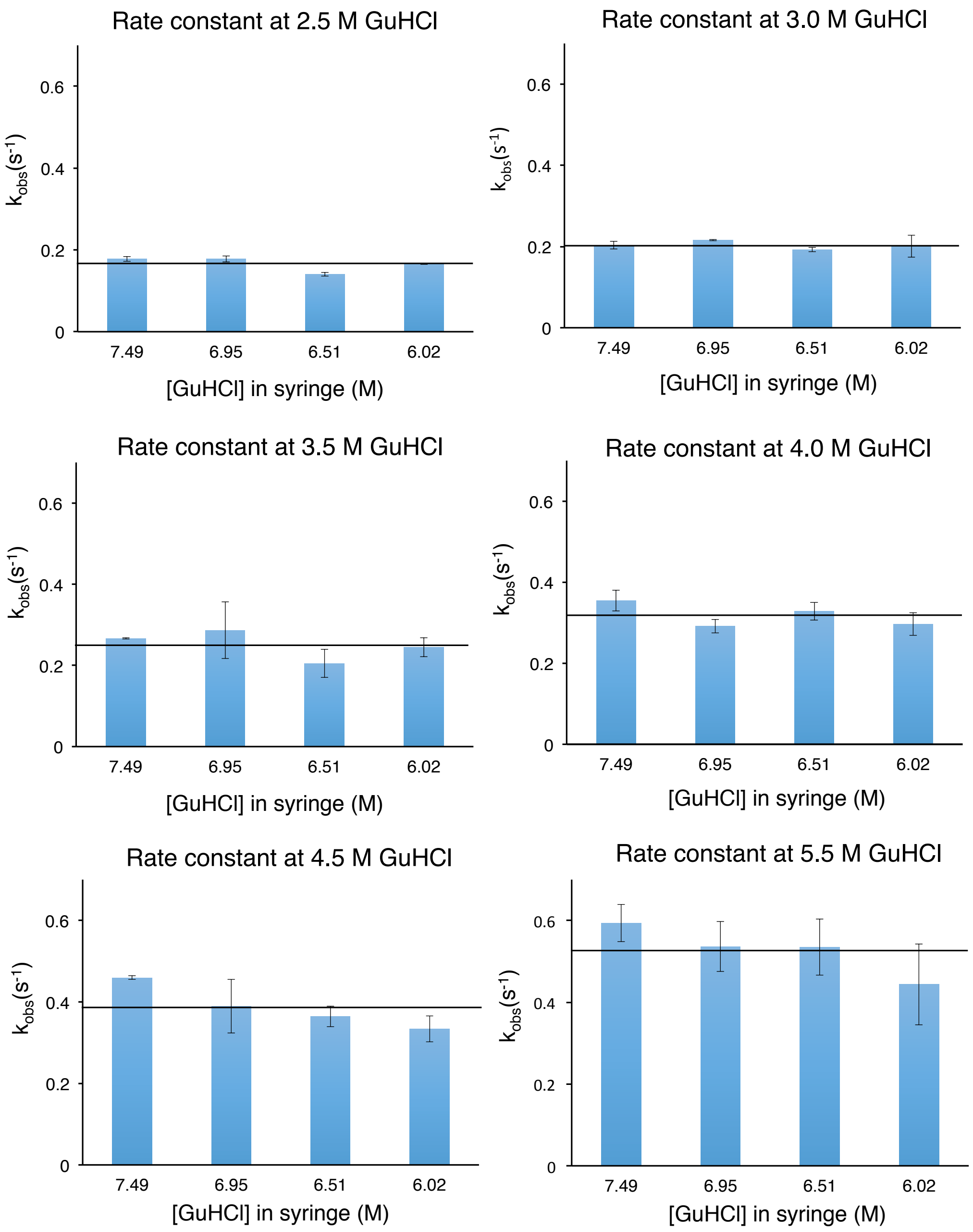

Fig. B Comparison of unfolding kinetic constants when the same final concentration of guanidine is reached using different protein:denaturant volume ratios. In almost all cases, the unfolding kinetic constants for AbpSH3 are very similar regardless of stock denaturant concentration. The horizontal line is the average unfolding rate for each set.

This highlights that whether the protein and denaturant solutions are mixed in a ratio of

2:1 or 1:10, the rapid mixing in the well still provides essentially equivalent kinetic information for the protein. At the start of a detailed study of new proteins, a shorter version of this test may be useful.

References

1. Walters J, Milam SL, Clark AC (2009) Practical approaches to protein folding and assembly: spectroscopic strategies in thermodynamics and kinetics. Meth Enzymol 455: 1–39.

2. Maxwell KL, Wildes D, Zarrine-Afsar A, Los Rios MA De, Brown AG, et al. (2005) Protein folding: defining a “standard” set of experimental conditions and a preliminary kinetic data set of two-state proteins. Protein Sci 14: 602–16.
