# Supplementary material for: Development and Application of a High Throughput Protein Unfolding Kinetic Assay
Source: PLoS One. 2016 Jan 8;11(1):e0146232. doi: 10.1371/journal.pone.0146232 (PMC4706425; doi:10.1371/journal.pone.0146232)
Supplement: S2 File — (PDF) [file pone.0146232.s006.pdf]

Supplementary File 2

Indirect folding parameters

The equilibrium m-value (which is a measure of the dependence of ΔG_u° on denaturant concentration) is the sum of the folding and unfolding m-value for 2-state proteins and as such, allows us to calculate an indirect folding m-value (equation 1b). Furthermore, the product of the equilibrium m-value and the D_{50} value (the molar concentration of denaturant when 50% of protein is unfolded) yields the free energy of unfolding, ΔG_u°, (equation 2), which can be converted to an equilibrium constant for unfolding according to equation 3 and this can be used to derive the folding kinetic constant according to equations 4 and 5. These equations are used in Table A.

$$m_{eq} = m_u - m_f \quad (1a)$$

where all units are kJ/mol M.

$$m_f = m_u - m_{eq} \quad (1b)$$

$$\Delta G_u^{\circ} = m_{eq}\, D_{50} \quad (2)$$

$$K_{eq\ (for\ unfolding)} = e^{\frac{-\Delta G^{\circ}}{RT}} \quad (3)$$

where R is the ideal gas constant (0.008314 kJ/mol K), and T is temperature in Kelvin.

$$K_{eq} = {k_u}/{k_f} \quad (4a)$$

where k_u is the unfolding kinetic constant and k_f is the folding kinetic constant.

$$k_f = {k_u}/{K_{eq}} \quad (4b)$$

Combining equations 6-8 yields 9

$$k_f = {k_u}\Big/{e^{\frac{-m_{eq}\, D_{50}}{RT}}} \quad (5)$$

Supplementary File 2

	m_{eq} (kJ/mol M) exp.	m_{eq} (kJ/mol M) lit.	D_{50} (M) exp.	D_{50} (M) lit.	ΔG°_{u} (kJ/mol) exp.	ΔG°_{u} (kJ/mol) lit.	k_f (s^{-1}) exp.	k_f (s^{-1}) lit.
**WT**	7.87 ± 0.07	6.86	1.64 ± 0.02	1.88	12.92 ± 0.04	12.89	12.0 ± 0.91	10.1 ± 1.1
**E7L**	7.46 ± 0.66	7.03	2.32 ± 0.07	2.57	17.31 ± 1.51	18.03	6.6 ± 0.48	17.3 ± 1.6
**V21K**	7.20 ± 0.44	n/a	2.02 ± 0.01	n/a	14.55 ± 0.93	14.48	15.6 ± 3.38	22.5 ± 0.6
**Triple**	6.49 ± 0.24	6.86	4.05 ± 0.002	3.92	26.29 ± 0.94	26.90	98.6 ± 26.02	34.5 ± 2.1
**HLL**	7.33 ± 0.80	n/a	3.12 ± 0.02	n/a	22.89 ± 2.49	n/a	37.0 ± 7.06	n/a

Table A. Summary of equilibrium data collected using the plate reader method and indirect folding rates. All proteins are WT or mutants of AbpSH3 except HLL. The literature m_{eq} and D_{50} values for V21K were omitted as they were only recorded using urea and not guanidine. "Lit" indicates the literature value and "exp" the experimental value measured in this study, "n/a" indicates that there is no literature value available for comparison. The error in k_f was calculated by differential analysis of equation 5 using the experimental uncertainties in k_u and ΔG°_{u}. As protein stability increases (smaller K_{eq}), the uncertainty in k_f correspondingly increases.

As can be seen in Table A, the folding constants obtained by this approach were on the same order of magnitude as literature values.
